# Immunosuppression Does Not Appear to Affect Echinococcal Cyst Viability as Confirmed by Immunoblotting Test: A Case Report in a Lung Transplant Patient

**DOI:** 10.4269/ajtmh.26-0403

**Published:** 2026-11-10

**Authors:** Raffaella Lissandrin, Ambra Vola, Domenica Federica Briganti, Sofia Frattola, Armanda Leka, Elena Seminari, Enrico Brunetti

**Affiliations:** ^1^ Division of Infectious Diseases I, Fondazione IRCCS Policlinico San Matteo, Pavia, Italy;; ^2^ WHO Collaborating Centre for Clinical Management of Cystic Echinococcosis, University of Pavia, Pavia, Italy;; ^3^ Microbiology and Virology Department, Fondazione IRCCS Policlinico San Matteo, Pavia, Italy;; ^4^ Regional Centre for Infectious Diseases (CEREMI), Lombardy Region, Italy;; ^5^ Pulmonology Unit, Fondazione IRCCS Policlinico San Matteo, Pavia, Italy;; ^6^ Department of Internal Medicine and Medical Therapy, University of Pavia, Pavia, Italy;; ^7^ Department of Clinical, Surgical Diagnostic and Pediatric Sciences, University of Pavia, Pavia, Italy

## Abstract

A 61-year-old Italian woman was diagnosed with a CE4 echinococcal cyst in the seventh hepatic segment. The cyst was managed conservatively (“watch and wait”) as per WHO guidelines. Serology for *Echinococcus granulosus* (ELISA) was negative. Five years later, in September 2025, the patient underwent bilateral lung transplant for chronic obstructive pulmonary disease. Induction with basiliximab, steroids, and treatment with tacrolimus and mycophenolic acid was started. Serology for *E. granulosus* (ELISA, rapid test [RT], and immunoblotting [IB]) remained negative, and the patient was managed expectantly. Serology with ELISA, RT, and IB remained negative at 0, 1, 3, and 6 months posttransplant. The cyst remained unchanged at all time points. Although anecdotal, this finding supports the hypothesis that unlike alveolar echinococcosis, immunosuppression has no effect on the viability of *E. granulosus* cysts. This has important clinical consequences. The American Society of Transplantation suggests treating patients for cystic echinococcosis before transplantation, and for adequate candidates, it recommends surgical removal with perioperative albendazole, an approach not supported by the literature. The need for treatment or surgical management of cystic echinococcosis in immunocompromised patients should be reconsidered.

## INTRODUCTION

Cystic echinococcosis is a rare occurrence in transplant patients, and it is often managed with extreme caution given the lack of data. In this paper, we describe a patient with an inactive echinococcal cyst of the liver who underwent lung transplant. No reactivation occurred during the follow-up. This suggests that immunosuppression does not affect cyst viability.

## CLINICAL CASE

A 61-year-old Italian woman was admitted to the intensive care unit of a tertiary hospital in northern Italy for severe respiratory distress owing to *Pasteurella multocida* lung infection. Invasive ventilation was required. The patient had severe emphysematous chronic obstructive pulmonary disease. An ultrasound (US) scan of the abdomen showed a single hepatic lesion 50 × 50 mm in diameter in the seventh segment, compatible with inactive CE4 cyst. As per WHO guidelines, no treatment was needed, and a US follow-up was planned.^1^ Serology for *Echinococcus granulosus* (ELISA) was negative.

In December 2023, her respiratory conditions worsened, and she was evaluated at our lung transplant center. As the cyst remained unchanged, only close follow-up with US and serology was planned.

In September 2025, 5 years after diagnosis of the cyst, the patient underwent bilateral lung transplantation with basiliximab and corticosteroid induction followed by maintenance immunosuppression with tacrolimus, mycophenolic acid, and corticosteroids. The cyst remained inactive on US 1 week posttransplant. Right pneumonia and urinary tract infection from *Escherichia coli* extended spectrum beta-lactamase occurred postoperatively, but once they were successfully treated, the patient was discharged.

On sonographic and serological follow-up at 1, 3, and 6 months, the cyst was unchanged (Figure 1). ELISA *Echinococcus* IgG (ELISA; NovaLisa^®^, Gold Standard Diagnostics, Dietzenbach, Germany), VIRapid^®^ HYDATIDOSIS (rapid test [RT]; Vircell, Granada, Spain), and *Echinococcus* Western Blot IgG (immunoblotting [IB]; LDBIO Diagnostics, Lyon, France) were performed at each time point as per the manufacturers’ recommendations (Table 1). Immunoblotting band patterns were evaluated to detect any changes in serological profile. All tests remained negative, and no IB bands were detected (Figure 2).

**Figure 1.**
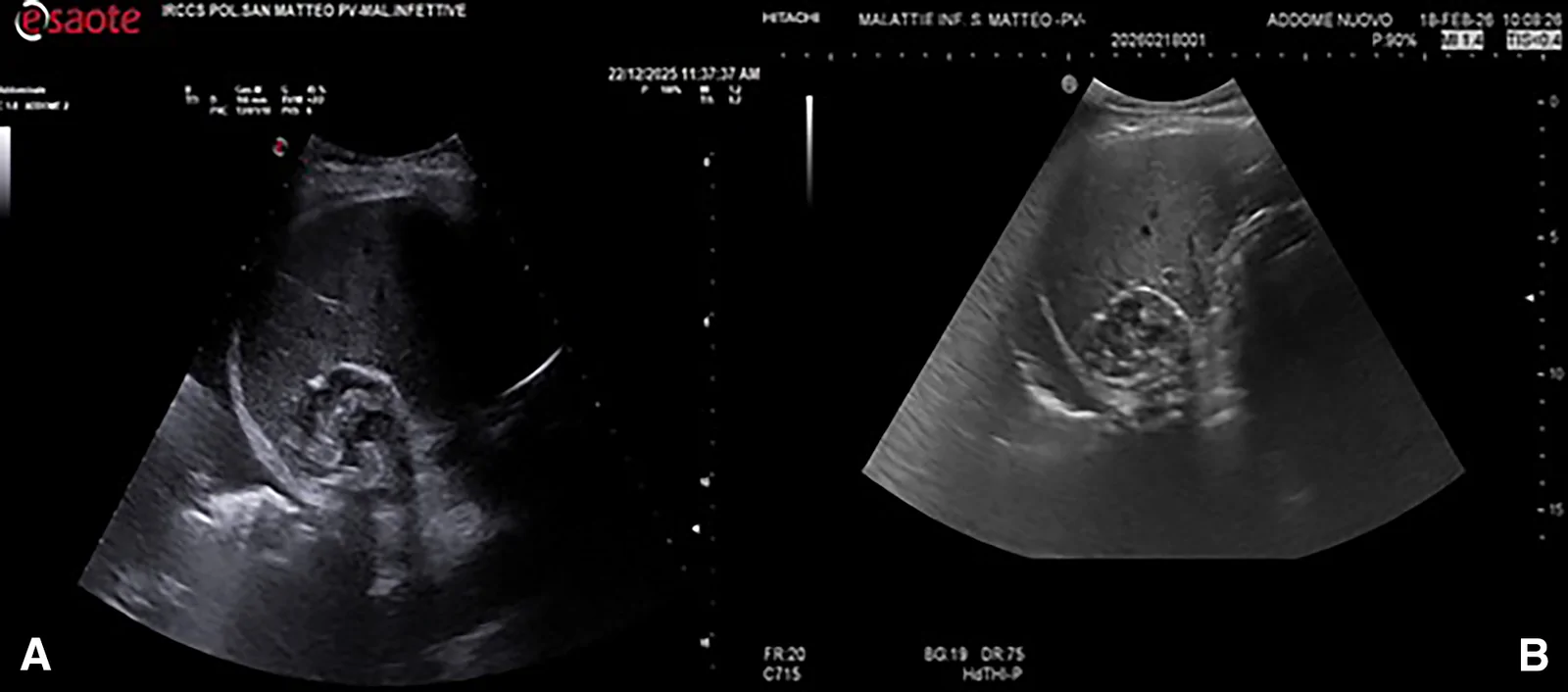
Posttransplant ultrasound imaging of the hepatica CE4 cyst in the seventh segment. (**A**) Three months. (**B**) Six months.

**Table 1:** Results of ELISA, rapid tests, and immunoblotting tests at different time points

Test	Pretransplant	Posttransplant	1 Month Posttransplant	3 Months Posttransplant	6 Months Posttransplant
ELISA	Neg	Neg	Neg	Neg	Neg
Rapid test	Neg	Neg	Neg	Neg	Neg
IB	Neg	Neg	Neg	Neg	Neg

IB = immunoblotting; Neg = negative.

**Figure 2.**
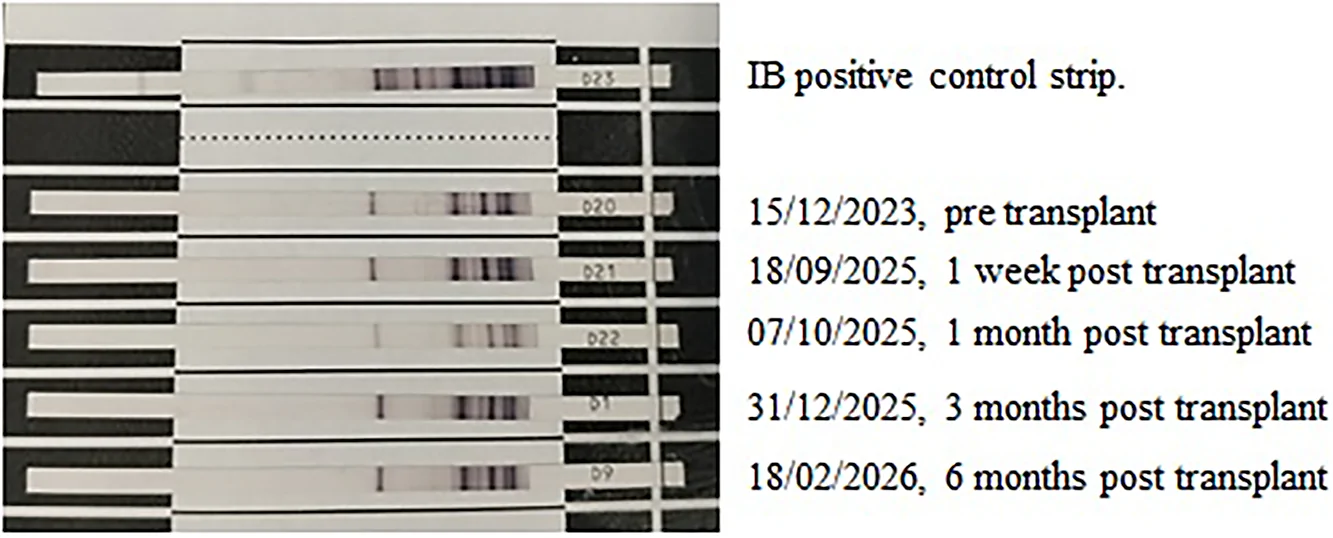
Immunoblotting (IB) strip test at different time points. No bands were detected at any time point pretransplant and posttransplant.

The patient is in good clinical condition at the time of writing.

## DISCUSSION

Cystic echinococcosis cysts go through different stages with different viability.^1^ Uncomplicated inactive cysts should be managed expectantly.^1^^–^^3^

A paper by our group showed that of 66 inactive uncomplicated CE4 and CE5 cysts without history of treatment, only 1 reactivated to a CE3b.^4^ No reactivation occurred in a cohort of 30 patients with untreated CE4 in Germany with a longer follow-up.^5^

However, these findings are from immunocompetent patients. No data exist on the behavior of inactive cysts in immunocompromised patients. This might justify the extreme caution in managing inactive cysts in immunocompromised patients. For example, the American Society of Transplantation suggests treating patients before transplantation and surgical removal with preoperative and postoperative albendazole (ABZ).^6^ Only two papers on cystic echinococcosis in transplant patients have been published. The first paper is from 1993, and it discussed a patient with a hepatic CE3b who underwent heart transplant.^7^ The second paper is from 2009, and it discussed a patient who developed a CE5 inactive cyst in the liver after renal transplant.^8^ Both were surgically treated for cystic echinococcosis after transplant, despite the stability of the cyst and the inactivity in the first^7^ and second^8^ papers, respectively.

In 2014, Mariconti et al.^9^ found that sera of patients with cystic echinococcosis cysts at different stages had distinctive band patterns using an experimental IB test based on human antigen. However, no specific bands were detected in all but one patient with inactive CE4/CE5 cysts. Mariconti et al.^9^ also tested sequential sera from one patient whose cyst changed from active (CE1) to transitional (CE3a) to inactive (CE4) in 24 months after ABZ treatment, and they observed a rapid change in band pattern from active to inactive stage, which points to serology mirroring cyst activity.^9^

Immunoblotting is considered the best single test to apply for the differential diagnosis of hepatic cystic echinococcosis, and it is still generally superior to running two different tests in parallel. Despite being the confirmatory serological test, its sensitivity in inactive stages remains low as around 50% of inactive cysts are seronegative. No data are available on the impact of immunosuppression on cyst activity and serological response. For these reason, we used both US to monitor the cyst and the IB bands pattern as a proxy for viability.

The cyst remained inactive (CE4) at US, and ELISA, RT, and IB remained negative, with no band detected with the IB test at each time point.

Although anecdotal, this case report suggests that immune suppression does not affect the viability of CE4 cysts in the liver, and it questions overtreatment of cystic echinococcosis in immunosuppressed patients.
